# Transcriptional shifts account for divergent resource allocation in feed efficient broiler chickens

**DOI:** 10.1038/s41598-018-31072-7

**Published:** 2018-08-27

**Authors:** Henry Reyer, Barbara U. Metzler-Zebeli, Nares Trakooljul, Michael Oster, Eduard Muráni, Siriluck Ponsuksili, Frieder Hadlich, Klaus Wimmers

**Affiliations:** 1 Institute for Genome Biology, Leibniz Institute for Farm Animal Biology (FBN), Wilhelm-Stahl-Allee 2, 18196 Dummerstorf, Germany; 2 Department of Farm Animals and Veterinary Public Health, Institute of Animal Nutrition and Functional Plant Compounds, University of Veterinary Medicine Vienna, Veterinaerplatz 1, 1210 Vienna, Austria; 3 Faculty of Agricultural and Environmental Sciences, University Rostock, 18059 Rostock, Germany

## Abstract

Considerable variation in feed efficiency (FE) has been observed in indigenous and selected meat-type chicken populations. Although this variation could be partially linked to extrinsic factors like diet, housing environment and microbiota, it further illustrates the existence of strong molecular mechanisms enabling the differential allocation of resources for various physiological processes. To further deepen the molecular basis of individual allocation capacity in male and female broilers, an RNA-seq experiment was conducted which based on a phenotyped chicken population divergent in FE. Transcriptional differences linked to FE were pronounced in intestinal and muscular tissue sites of male animals. Specifically, signalling pathways of farnesoid X receptor (FXR) and retinoid X receptor (RXR) might contribute to mediate individual FE. The transcriptional profiles suggested *ACSBG2* (muscular lipid utilisation), *ASBT* (intestinal bile salt transport), *CLEC2B* (natural killer cell activation), *HMGCS2* (jejunal, duodenal and muscular ketogenesis), and *SCARB1* (jejunal lipid uptake) as potential mediators driving FE. Results indicate that improvements in FE exploit shifts in resource allocation which might occur at the expense of general immune responsiveness in high efficient male chickens. Consequently, to further improve FE traits and to explore causative molecular patterns, effects originating from sex-dimorphism in chickens need to be taken into consideration.

## Introduction

An efficient conversion of nutrients into live weight is an important aspect of livestock production to reduce the environmental footprint and to increase the overall performance towards a sustainable intensification of agri-food production^1^. The bird’s capability for an efficient utilization of nutrients culminates in the individual’s feed efficiency (FE) which can be expressed in different ways in broilers. Most common measurements such as feed conversion ratio (FCR) and residual feed intake (RFI) consider records of energy consumption like feed intake (FI) and performance measures such as body weight gain (BWG). These proxies contribute to describe the complex molecular basis of FE traits^2^. Therefore, several genetic studies of FE- tested broiler populations (e.g.^3,4^) and broiler lines divergently selected for FE traits (e.g. digestive efficiency^5^) revealed a steadily growing number of genomic features influencing FE, FI, and BWG. Moreover, recent targeted and holistic transcriptomic analyses provide first insights into pathways and biological functions contributing to individual differences in FE of meat-type chickens. At the same time, these studies are indicative for the tissue-specific plasticity of expression patterns induced during different developmental stages (e.g.^6,7^) and under varying environmental conditions^8^. The mitochondrial efficiency of energy production has been identified as one of the major molecular mechanisms driving FE in muscle tissue of meat-type chickens^9^. In this context, AMP-activated protein kinase (AMPK) was suggested to play a considerable role via sensing the cellular energy status and balance metabolic activities^10,11^. Other main processes which were found to be affected comprise genes involved in the function and structure of the digestive system^5^, in the regulation of appetite as well as in lipid metabolism and transport^12^. Moreover, shifts in the utilization of distinct metabolic pathways, such as the shift from purine biosynthesis pathway to the purine salvage pathway were recently suggested as energy-saving strategies to improve FE^13^. Thus, organismal resource allocation is considered to be of central relevance to improve FE. In this respect, the energy demand that is required by the immune system was reported to be equal to 9% of the nutrient consumption^14^. Especially, the innate immunity is an energetically expensive process which is supposed to be suppressed in meat-type chickens selected for high growth rates^15^. Indeed, selection for high production efficiency might co-evolve behavioral, physiological, and immunological concerns as reviewed elsewhere^16^.

Despite long-term efforts to improve phenotypic traits in chickens, even individuals within highly selected broiler lines still vary considerably in their FE, nitrogen excretion and growth performance under controlled environmental conditions, as previously shown by Metzler-Zebeli *et al*.^17^ Building upon the study of Metzler-Zebeli *et al*. (2016), extreme male and female chickens with regards to RFI were selected here and analysed via RNA-seq to identify tissue-specific transcriptional responses. Thus, genome-wide transcriptome profiling comprised muscle tissue (i.e. breast muscle; *M*. *pectoralis*) as the major consumers of energy and different parts of the small intestine (i.e. duodenum, jejunum, ileum) facilitating digestion and absorption of nutrients.

## Results

The study investigates transcriptional patterns of muscular and intestinal tissues in meat-type chickens considerably divergent in their RFI. With regard to the widely unclear molecular mechanisms driving FE and the pronounced sex dimorphism in chickens, the objectives of this study were to identify sex-specific metabolic routes and molecular features contributing to FE.

### Measurements of feed efficiency traits

The analyses of all sampled chickens with divergent RFI (n = 36) revealed differences in mean RFI values for both males (−183 g vs. 303 g; p < 0.001) and females (−195 g vs. 197 g; p < 0.001)^18^. In Table 1, data on RFI and FCR of the twelve animals selected for comprehensive transcriptomic analyses representing the low and high RFI groups of the whole trial population are summarized. Here, significant differences in RFI and FCR values reflect the divergent FE in animals of both sexes (Table 1). Moreover, low FE female and low FE male chickens showed significant differences in their RFI (203 ± 31 g vs. 431 ± 48 g; p = 0.018).

**Table 1:** Mean, standard error and statistics of feed efficiency (FE) indicators comprising residual feed intake (RFI in g) and feed conversion ratio (FCR in g/g, in brackets) between experimental groups (n = 12).

	High FE (female)	High FE (male)	Low FE (female)	Low FE (male)
High FE (female)	−212 ± 37 (1.45 ± 0.03)	0.885	<0.001	<0.001
High FE (male)	0.736	−254 ± 45 (1.38 ± 0.02)	<0.001	<0.001
Low FE (female)	0.006	0.003	203 ± 31 (1.60 ± 0.02)	0.018
Low FE (male)	0.001	<0.001	0.273	431 ± 48 (1.63 ± 0.03)

The diagonal depicts mean ± SE of RFI and FCR (in brackets). Above the diagonal p-values for the comparison of RFI values are displayed; below the diagonal p-values for the comparison of FCR values are displayed.

### Differentially abundant genes in muscular and intestinal tissues

**Figure 1:**
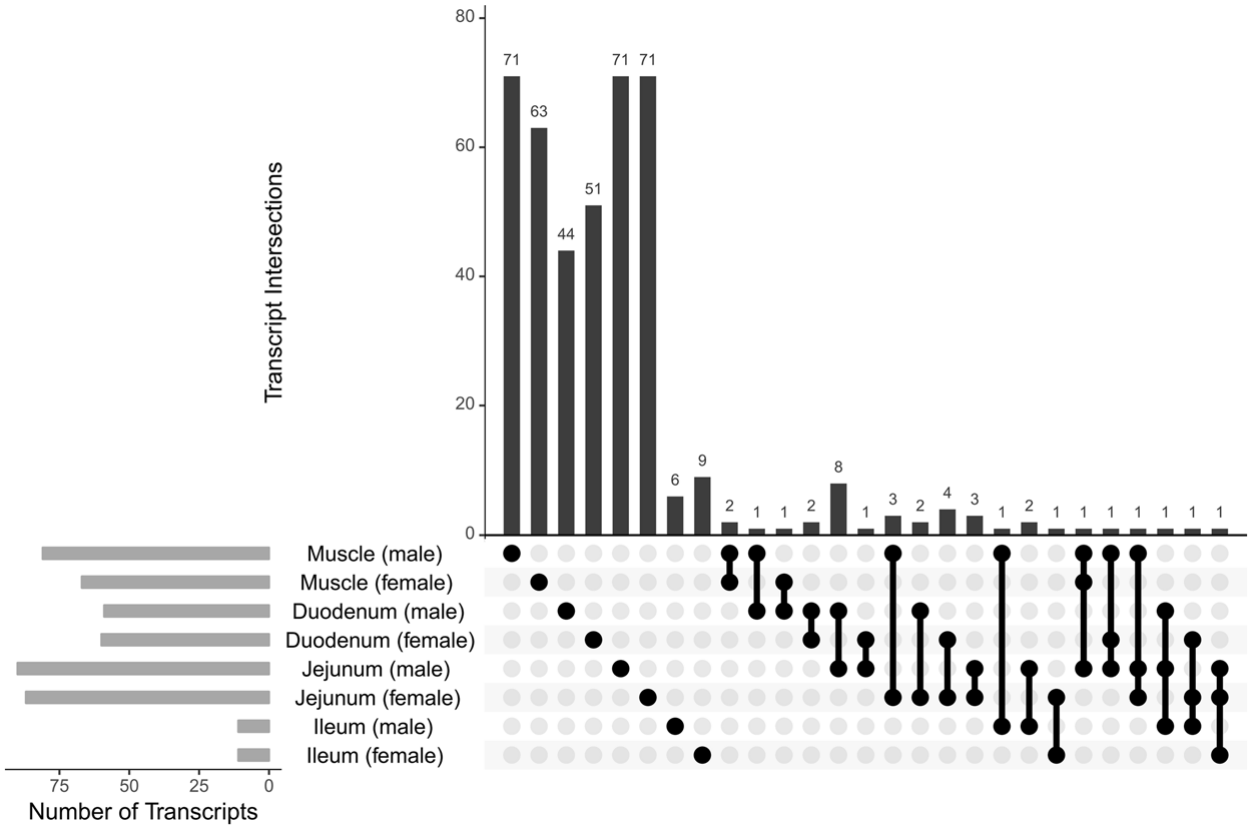
Number of differentially abundant transcripts (bottom left) and intersections of transcripts (upper right) identified by RNA-seq for divergent feed efficiency in and between different tissues for male and female broilers. Overlapping genes between different tissues are indicated by connected dots.

RNA sequencing yielded an average of 80 million single reads for each sample. The subsequent mapping of reads against the chicken reference genome resulted in a concordant pair alignment rate of 87.6 ± 1.8%. The following statements refer to 44 out of 48 samples that met the quality control criteria. Considering the significance threshold (p < 0.001 and q < 0.25) the following number of genes were found to be differentially abundant between high and low FE groups: 67 (female) and 81 (male) in breast muscle; 60 (female) and 59 (male) in duodenum; 87 (female) and 90 (male) in jejunum; 11 (female) and 11 (male) in ileum (see Supplementary Table S1). Numbers of differentially abundant transcripts as well as their intersections within and between experimental groups are visualized in Fig. 1. Consistent across both sexes the proenkephalin encoding gene *PENK* was significantly higher abundant in breast muscle of high FE animals. In contrast, the acyl-CoA synthetase bubblegum family member 2 encoding gene *ACSBG2* and a novel gene named ENSGALG00000033498 were found to be differentially abundant in males and females. Overlapping results were also present for the transcript abundance of *KCNK17* (potassium two pore domain channel subfamily K member 17) and ENSGALG00000045251 in duodenal samples. For the jejunum, genes related to metabolic pathways i.e. 6-phosphofructo-2-kinase/fructose-2,6-biphosphatase 3 (*PFKFB3*), glucose-6-phosphatase catalytic subunit (*G6PC*), apical sodium-dependent bile salt transporter D (*ASBT*/*SLC10A2*) and solute carrier family 10 member 2 (*SLC10A2*) were revealed as significantly differentially abundant in male and female chickens with opposite expression patterns in both sexes. Interestingly, the transcript encoded by the novel gene ENSGALG00000033116 was consistently less abundant in all examined intestinal parts of high FE male broilers compared to low FE male chickens. Based on the annotation of the human orthologue, this gene is predicted to encode a C-type lectin.

For genes found to be more abundant in high FE compared to low FE birds, indicated by positive fold changes (FC), highest FC differences were assigned to *HMGCS2* (FC = 4.3; duodenum female), ENSGALG00000033498 (FC = 4.1; jejunum male) and *ST6GALNAC1* (FC = 4.0; duodenum male). Highest negative FC (high FE < low FE) were identified for ENSGALG00000019325 (FC = −9.7; duodenum male), *AMY2A* (FC = −7.2 duodenum female) and ENSGALG00000019845 (FC = −7.1; muscle male).

### Analyses of pathways

The integration of RNA-seq results revealed significantly enriched canonical pathways (adjusted p < 0.05), which are presented in Table 2. In general, the comparison of high and low FE males showed a higher number of enriched pathways compared to the same comparison in females. In the breast muscle of male chickens, the highest significantly enriched pathways were the ‘FXR/RXR activation’ (−log_10_[FDR] = 23.3) and ‘LXR/RXR activation’ (−log_10_[FDR] = 21.1). These pathways are assigned to the ‘nuclear receptor signalling’ pathway category, which is further represented by ‘PXR/RXR activation’ and ‘LPS/IL-1 mediated inhibition of RXR function’ among the significantly enriched pathways in muscle tissue. The same canonical pathways, assigned to the ‘nuclear receptor signalling’ category, were revealed in the comparison of jejunum samples of high and low FE male chickens.

**Table 2:** Canonical pathways in breast muscle, duodenum, jejunum and ileum tissue of feed efficiency-divergent male and female broilers.

Tissue	Sex	Ingenuity Canonical Pathway^1^	−log10(FDR)	Molecules
Breast muscle	male	FXR/RXR Activation	23.3	AGT, AHSG, ALB, AMBP, APOA4, APOB, APOH, FETUB, FGA, G6PC, GC, HPX, KNG1, ORM1, **PLTP**, RBP4, SERPINA1, VTN
		LXR/RXR Activation	21.1	AGT, AHSG, ALB, AMBP, APOA4, APOB, APOH, FGA, GC, HPX, KNG1, LPA, ORM1, **PLTP**, RBP4, SERPINA1, VTN
		Acute Phase Response Signalling	12.8	AGT, AHSG, ALB, AMBP, APOH, F2, FGA, FGB, HPX, HRG, ITIH2, ORM1, RBP4, SERPINA1
		Coagulation System	8.9	F2, F9, FGA, FGB, KNG1, SERPINA1, SERPINC1
		Intrinsic Prothrombin Activation Pathway	7.8	F2, F9, FGA, FGB, KNG1, SERPINC1
		Clathrin-mediated Endocytosis Signalling	5.6	ALB, APOA4, APOB, F2, ITGB2, LPA, ORM1, RBP4, SERPINA1
		Extrinsic Prothrombin Activation Pathway	4.9	F2, FGA, FGB, SERPINC1
		IL-12 Signalling and Production in Macrophages*	4.7	ALB, APOA4, APOB, LPA, ORM1, RBP4, SERPINA1
		LPS/IL-1 Mediated Inhibition of RXR Function	2.1	ACSBG2, CYP2C19, FABP1, HMGCS2, **PLTP**
		PXR/RXR Activation	2.0	CYP2C19, G6PC, HMGCS2
		Autophagy	1.8	**CTSC**, **CTSH**, **CTSS**
		Nicotine Degradation III*	1.6	ADH7, CYP2C19
		Granulocyte Adhesion and Diapedesis	1.4	CLDN10, **CSF3R**, **ITGB2**
	female	—		
Duodenum	male	Estrogen Biosynthesis	1.3	AKR1B15, CYP51A1
		Retinoate Biosynthesis I	1.3	RDH11, SDR9C7
		Nicotine Degradation III*	1.3	CYP51A1, UGT2B15
		Superpathway of Cholesterol Biosynthesis	1.3	CYP51A1, FDPS
	female	Superpathway of Cholesterol Biosynthesis	1.3	**HMGCS2**, **MSMO1**
Jejunum	male	LPS/IL-1 Mediated Inhibition of RXR Function	2.9	CYP4A11, **GSTA1**, GSTA3, HMGCS2, LBP, NR0B2, **SCARB1**
		FXR/RXR Activation	2.6	FGF19, G6PC, NR0B2, **SCARB1**, **SLC10A2**
		PXR/RXR Activation	2.5	G6PC, **GSTA1**, HMGCS2, NR0B2
	female	—		
Ileum	male	Crosstalk between Dendritic Cells and Natural Killer Cells	3.3	CD69, **HLA-C**, HLA-G
		Antigen Presentation Pathway*	2.9	**HLA-C**, HLA-G
	female	—		

^1^Pathways indicated by less than two molecules were discarded. Pathways represented by the same set of molecules were excluded with the highest significantly enriched canonical pathway shown (indicated by an asterisk*).

The genes highlighted in bold were higher abundant in the high FE group compared to low FE group.

The analyses of breast muscle further indicated an enrichment of pathways assigned to the cellular immune response such as ‘acute phase response signalling’ and ‘IL-12 signalling and production in macrophages’ in male chickens. In the duodenum, the ‘superpathway of cholesterol biosynthesis’ was revealed to be altered between high and low FE animals in both sexes. In the ileum, affected pathways in males were based on orthologues genes of the human leukocyte antigen (HLA) system and were assigned to the ‘cellular immune response’ category (‘Crosstalk between Dendritic Cells and Natural Killer Cells’ and ‘Antigen Presentation Pathway’. It should be noted that pathways identified for duodenum and ileum are represented by only 3 or fewer molecules. No significantly enriched pathways were obtained for the analysis of breast muscle, jejunum and ileum tissue of female chickens.

### Analyses of biofunctions

Biofunctions retrieved from IPA were analysed to deduce consequences as well as tissue- and sex-specific contributions in the context of FE. Biofunctions which were predicted to be significantly activated (z-score > +2) or inactivated (z-score < −2) in high FE chickens are shown in Table 3. Altered biofunctions were exclusively indicated for breast muscle and jejunum samples of male chickens. For breast muscle, the enriched themes were related to cell signalling and interaction, inflammatory response, metabolism and molecular transport. With the exception of the ‘inflammation of absolute anatomical region’ function, biofunctions were predicted to be inactivated in high FE chickens compared to low FE chickens. Interestingly, all indicated biofunctions rely on *AGT* and *F2*, coding for the angiotensinogen precursor and coagulation factor 2, respectively. Consequently, the biofunction related to angiogenesis was predicted to be activated in breast muscle of high FE male chickens, albeit its corresponding z-score was below the significance threshold (z-score = +1.85).

**Table 3:** Enriched biological functions with predicted activation state in breast muscle and jejunum of FE-divergent broilers.

Tissue (sex)	Molecular theme	Ingenuity Biofunction	Molecules
Breast muscle (male)	Cell signalling	quantity of Ca^2+^	AGT, F2, **HSPA5**, KNG1, LPA, ORM1, **SPP1**
	Cell-to-cell signalling and interaction	activation of cells	AGT, ALB, APOB, APOH, **CTSH**, **CTSS**, F2, FGA, GC, HLA-G, HRG, **ITGB2**, KNG1, **PENK**, PLTP, RBP4, SERPINA1, **SPP1**, VTN
		activation of antigen presenting cells	AGT, APOH, **CTSH**, **CTSS**, F2, GC, KNG1, RBP4, VTN
		activation of macrophages	AGT, APOH, F2, GC, KNG1, VTN
		activation of leukocytes	AGT, APOH, CTSH, CTSS, F2, GC, HLA-G, ITGB2, KNG1, PENK, RBP4, SPP1, VTN
	Inflammatory response	**inflammation of absolute anatomical region**	AGT, ALB, AMBP, APOA4, APOB, APOH, **CSF3R**, **CTSS**, F2, FABP1, FGA, GC, HPX, **HSPA5**, **ITGB2**, **SPP1**
	Lipid metabolism	release of eicosanoid	AGT, ALB, F2, KNG1, SERPINC1
		fatty acid metabolism	ACSBG2, AGT, ALB, APOA4, APOB, APOH, **CTSS**, F2, FABP1, GC, KNG1, **PLTP**, RBP4, SERPINC1, VTN
		synthesis of fatty acid	AGT, ALB, APOA4, APOB, F2, KNG1, SERPINC1, VTN
		synthesis of eicosanoid	AGT, ALB, F2, KNG1, SERPINC1, VTN
	Molecular transport	transport of molecule	AGT, ALB, APOA4, APOB, APOH, **CTSS**, F2, FABP1, FGA, FGB, G6PC, GC, GJB2, HPX, **HSPA5**, KNG1, **PLTP**, RBP4, **SLC37A2**
	Nucleic acid metabolism	biosynthesis of cyclic nucleotides	AGT, F2, KNG1, LPA, **SPP1**
Jejunum (male)	Carbohydrate metabolism	**quantity of monosaccharide**	FGF19, G6PC, **HSPA5**, NR0B2, **PFKFB3**, **SCARB1**
		**quantity of carbohydrate**	ADA, FGF19, G6PC, **HSPA5**, LBP, NR0B2, **PFKFB3**, **SCARB1**
	Endocrine system development and function	**concentration of hormone**	ABHD6, FAAH, NEIL1, NR0B2, PLIN1, **SCARB1**, TSC22D3
	Lipid metabolism	**concentration of lipid**	ABHD6, ADA, FAAH, FGF19, G6PC, **GSTA1**, LBP, NEIL1, NR0B2, PLIN1, **SAA2-SAA4**, **SCARB1**, **SLC10A2**, TSC22D3
		**concentration of acylglycerol**	ABHD6, FAAH, FGF19, G6PC, NEIL1, NR0B2, **SAA2-SAA4**, **SCARB1**

The Biofunctions highlighted in bold were predicted to be activated (z > +2), biofunctions in normal font were predicted to be inactivated (z < −2) in high FE group compared to the low FE group. Involved molecules highlighted in bold were higher abundant in the high FE group compared to the low FE group.

In jejunum samples of FE-divergent male chickens, analysis revealed a predicted activation of molecular themes related to carbohydrate and lipid metabolism as well as endocrine system development. Specifically, IPA results predict a higher quantity of monosaccharides and a higher concentration of lipids in jejunal samples of high FE chickens compared to low FE chickens. Central molecules which contributed to all biofunctions were an orphan member of the nuclear receptor superfamily encoded by *NR0B2* and the scavenger receptor class b member 1 encoded by *SCARB1*. The former was less abundant and the latter was higher abundant in high FE males compared to low FE males.

### Verification of RNA-seq results

**Figure 2:**
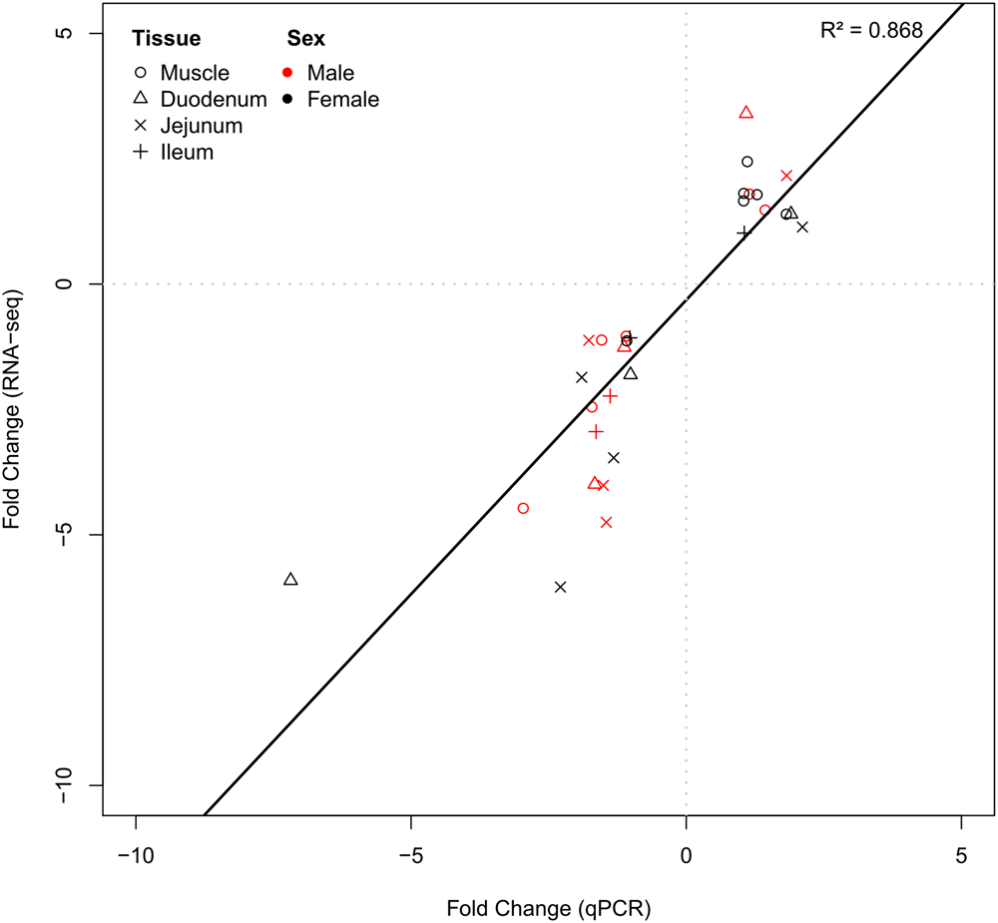
Validation of RNA-seq results by quantitative real-time PCR (qPCR) of selected transcripts. Fold changes between experimental groups in qPCR are plotted against values obtained from RNA-seq. The Pearson correlation coefficient is represented by R^2^.

The validity of the RNA sequencing experiment and subsequent statistical data analysis was verified by comparing results obtained via RNA-seq and qPCR (Fig. 2, Supplementary Table S3). The magnitude of fold changes in mRNA abundances was reproducible with both approaches. The correlation of results was as significant and revealed a correlation coefficient of 0.868 (see also Supplementary Table S3). Consequently, the qPCR analyses support the reliability of RNA-seq results.

## Discussion

In the context of divergent RFI as an indicator for FE, the study revealed pronounced differences between male and female broilers at the phenotypic and transcriptional level. According to the data of the whole trial population, the phenotypic differences comprised a higher final body weight, a better FCR and higher daily dry matter excretion of males compared to females^18^. Similarly, sex-specific differences were observed for various production traits across different meat-type chicken lines^19,20^. In the studied population, the range of RFI values was higher in males than in females^18^. Accordingly, the subset of birds with most extreme phenotypes regarding RFI used for transcriptome analyses showed significant differences of RFI values between low FE males and low FE females. Transcriptomic profiles observed for the analysed tissues of high and low FE chickens differed considerably between the two sexes. It is also worth mentioning that a lower number of differentially abundant transcripts were identified in ileum samples compared to the other tissues analysed. This might represent the major importance of the proximal parts of the small intestine for the FE of pigs in concentrate-based diet. These sex and tissue-specific peculiarities were also reflected in the number of significantly affected canonical pathways and biofunctions in the comparisons between the different groups. Hence, despite extensive performance testing and novel insights into quantitative genetics contributing to improved FE, results implicate that certain sex-specific molecular routes and biofunctions might be targeted in the context of FE in broilers.

The most prominent category of enriched pathways identified in the jejunum and breast muscle of male broilers was related to the nuclear receptor signalling. This category comprised pathways triggered by retinoid X receptor (RXR), farnesoid X receptor (FXR or NR1H4) and liver X receptor (LXR) activation. These receptors are present in numerous tissues with highest expression levels of FXR and LXR in liver, intestine and kidney^21^. Signalling pathways downstream of these receptors influence different developmental and metabolic processes including cholesterol and bile acid homeostasis^22^, which might contribute to improve FE in broilers. Indeed, an altered lipid, cholesterol and bile acid metabolism including effects mediated via FXR/RXR signalling were previously presented as one of the key molecular mechanisms driving differences of FE in the duodenum of male meat-type chickens divergently selected for RFI^12^. Similar pathways were found to be affected at the level of the muscle and jejunum in the current study. Based on the altered biofunctions in muscle tissue, these differences were suggested to result in the decrease of molecular transport processes and fatty acid metabolism in high FE male chickens. In this context, the *ACSBG2* encoded acyl-CoA synthetase plays an important role for the regulation of lipid metabolism in chickens by initiating the intracellular decomposition or conversion of long-chain fatty acids (mainly C18:1 and C18:2)^23^. Although expression of human and murine *ACSBG2* was reported to be specific for testis and brainstem^24^, the gene appeared to be among the highest expressed genes in chicken muscle of both sexes (see Supplementary Table S1). Interestingly, the FC of *ACSBG2* in muscle tissue of males and females was shown to be in opposite directions. Therefore, this gene might contribute to known sex-specific differences in FE and fat traits in chickens^19^. The lower abundance of transcripts associated with apolipoproteins such as *APOA4*, *APOB* and *APOH* in muscle tissue of high FE male chickens indicates for a reduced muscular presence of lipoproteins linked to a putative decrease in intramuscular fat, as previously described^25^. Consequently, serum cholesterol levels tended to be lower in high FE male broilers and were proposed as a valuable indicator for FE by Metzler-Zebeli *et al*.^18^.

In the jejunum of male broilers, pathways dedicated to nuclear receptor signalling (FXR, RXR, PXR) seem to mediate effects on the intestinal absorption of bile salts as well as dietary cholesterol and triglycerides. Specifically, genes like *SCARB1* and *ASBT* were found to be more abundant in the jejunum of high FE male birds, while *NR0B2*, which mediates the FXR effects^26^ was less abundant. *SCARB1* encodes a plasma membrane receptor for lipoproteins which is involved in the absorption of cholesterol from the diet and, thus, is known to differ in abundance depending on the dietary level of lipids in humans^27,28^. *ASBT* is the primary intestinal bile salt transporter in the intestine^29^ and is negatively regulated by FXR^26^. Interestingly, the analyses of jejunal samples of female broilers revealed that *ASBT* was significantly less abundant in high FE animals. This might implicate sex-specific differences regarding the jejunal bile acid metabolism. Based on these results, it is suggested that high FE male chickens had an increased absorption of dietary cholesterol and a more efficient recovery of bile acid compared to low FE broilers. Moreover, it is known that an intestinal FXR activation is linked to a reduced *de novo* synthesis of fatty acids in other tissues^30^, which is in line with the predicted inactivation of fatty acid synthesis in muscle tissue of high FE male broilers.

At the same time, altered transcript abundances in muscle (e.g. *G6PC*, *HMGCS2*) and jejunum (e.g. *G6PC*, *PFKFB3*) of male chickens affect metabolic routes of carbohydrate metabolism. Specifically, effects observed in the muscle of male broilers indicate a reduced ketogenesis in high FE animals which were likely mediated via mitochondrial HMG-CoA synthase (encoded by *HMGCS2*), as a central regulator of ketogenesis^31^. This might imply for a metabolic demand to allocate sufficient amounts of carbohydrates into muscle tissue of high FE broilers. Biofunctions pointing to an increased availability of monosaccharides and lipids in the jejunum derived by a lower jejunal abundance of *HMGCS2* in high FE males support this suggestion. Interestingly, chickens selected for reduced RFI values showed an increased abundance of *HGMCS2* in the duodenum^12^. The same transcriptional alterations were found in the comparison of duodenal samples between high and low FE chickens in the current study, albeit reaching significance exclusively in females. In conclusion, the findings promote a central role of nuclear receptor signalling for FE in different tissues affecting the utilization of lipids and carbohydrates.

Although considerable differences were observed between high and low FE chickens of both sexes, there were no signs for major differences in muscle development and cellular energy homeostasis at the transcriptional level. Especially, mitochondrial efficiency and AMPK signalling were recently proposed as important biological processes involved in improving FE^9,10^, but were unaffected in the analysed FE-divergent broilers. However, commercial broiler lines are under selection for improved body weight gain and FE for many generations leading to a favoured genetic pattern regarding these traits^3^. Thus, minor alterations of different biological pathways might culminate in the observed differences between high and low FE broilers. As recently described in livestock species, trade-offs between performance traits and immune response have to be considered as one important factor contributing to individual differences in productivity^32,33^. With this regards, all tissues examined in the current study are known to contribute to divergent FE via molecular factors involved in the modulation of immune responses^34^. Thus, biofunctions derived from the comparison of breast muscle expression profiles of high and low FE male chickens as well as pathways enriched for the comparisons of breast muscle and ileum indicated for modest differences in immune system functions. Additionally, alterations observed at the level of the lipid metabolism might also have implications on cell signalling and inflammatory responses, for instance, via the predicted decrease in eicosanoid synthesis and release in muscle tissue of high FE male chickens^35^. This is in line with ongoing discussions regarding the energy demand to maintain immune system functions^14–16^ and possibilities to modulate the immune system in chickens^36^. Specifically, the results of the current study argue for shifts in favour of a disease resistance strategy of low FE broilers which might employ the innate immune response providing a broad-spectrum protection while being energetically expensive^14,37^. In contrast, high FE chickens seem to rely on tolerance mechanisms promoting specific immune reactions^38^. This is supported by the predicted decrease in the activation of macrophages in the muscle of high FE male chickens, which at the same time showed activated biological functions of inflammatory response. Moreover, Metzler-Zebeli *et al*.^18^ reported a significant increase in the lymphocyte fraction and a trend towards a decreased heterophile fraction of white blood cells in high FE male broilers compared to low FE male birds of the same population. In this context, ENSGALG00000033116 (orthologue of human *CLEC2B*) was consistently found to be lower abundant in all three analysed parts of the small intestine of high FE chickens, albeit reaching statistical significance exclusively for the comparison of male broilers. In humans, *CLEC2B* is located in the natural killer (NK) cell gene complex and is known to encode a cell activation antigen presented by NK cells, which are an important component of the innate immune system^39,40^. Hence, the intestinal lower abundance of *CLEC2B* in high FE chickens might represent an indicator for a reduced activation of the innate immune system. However, due to the complex contributions to differences in FE, other aspects like FE-related differences in the intestinal microbiota composition^41^ need to be considered modulating the host’s immune capacity at the transcriptional level (e.g.^42^).

## Conclusion

Phenotype differences in RFI values were reflected by pronounced sex-dimorphic transcriptional patterns. Indeed, the RNA-seq analyses of muscle and intestine tissues in broiler chickens selected for divergent RFI revealed cholesterol and bile acid metabolism as well as immunomodulatory aspects as molecular drivers of FE in male chickens. Moreover, results indicate that improvements of FE exploit shifts in resource allocation which might occur at the expense of general immune responsiveness in high efficient male chickens. Hence, data suggest that breeding strategies have also to focus beyond improvements in FE traits towards maintenance of phenotypes balancing weight, performance and immune competence.

## Material and Methods

### Animals, housing, feed efficiency assessment and tissue sampling

The conducted chicken trial was approved by the animal ethics committees of the University of Veterinary Medicine (Vienna, Austria) and the national authority according to paragraph 26 of Law for Animal Experiments, Tierversuchsgesetz 2012 (GZ 68.205/0131-II/3b/2013). Animal trials were performed in three independent batches with at least 52 birds per batch. Chickens were obtained from commercial hatcheries. The experimental design was balanced for sex and comprised 157 birds (79 males and 78 females). Housing and feeding conditions have been previously described^17,18^. In brief, one-day-old Cobb500FF birds (Cobb-Vantress, Siloam Springs, AR, USA) were housed in groups of four birds for 7 days. Afterwards, they were individually penned in cages until the end of the experimental period. Chickens were checked daily for health status and welfare issues; signs of illness led to the exclusion of animals for further analysis. Common diets based on corn and soybean meal were fed during starter (days 1–10), grower (days 11–21), and finisher periods (days 22–42) as previously described^17,18^. *Ad libitum* access to fresh water was provided. Individual FI was recorded daily. Body weight of all chickens was recorded weekly (d 1, 7, 14, 21, 28 and 35). To calculate overall RFI values, body weight was further measured at d 36. For RFI calculation, data for net total FI, metabolic mid-weight and total BWG between day 7 and 36 were analysed using a nonlinear mixed model (SAS Stat Inc., version 9.2, Cary, NC) as previously described^17,18^. Accordingly, for each batch and sex, the 3 chickens with the lowest RFI (high FE) and the 3 chickens with the highest RFI (low FE), were selected at day 36 of life. Per batch, twelve RFI-divergent animals (3 low RFI, 3 high RFI; both sexes) were dissected on four consecutive days. In total, 36 birds were sampled. Gut tissue samples (1 cm^2^) were collected from duodenum, jejunum and ileum. Duodenum samples were taken distal to the Flexura duodeni. The jejunum was sampled at the distal end close (5 cm) to the Meckel’s diverticulum. Ileum samples were taken approximately 17 cm proximal to the ileo-caecal junction. Muscle samples (*M*. *pectoralis)* were collected from the bottom half of the left breast. Gut and breast muscle samples were immediately cleaned in phosphate-buffered saline, blotted dry on paper towel, in case of the muscle samples cut into small cubes, snap-frozen in liquid nitrogen, and stored at −80 °C until use.

### RNA isolation and RNA-seq

A subset of animals divergent in RFI values (high RFI, low RFI) and representative for the trial population was selected for transcriptome analysis (from batch three; *n* = 12). A balanced design accounting for sex and slaughter day was applied. Descriptive statistics for all chickens were previously provided by Metzler-Zebeli *et al*.^17,18^. For selected animals, individual FE measurements (RFI and FCR) were compared using a linear mixed model (R Project for Statistical Computing, version 3.3.1, lmerTest package). The model included the interaction of sex (male, female) and RFI group (high, low) as fixed effect and the day of slaughter as random effect. P-values were adjusted for multiple comparisons using the Tukey-Kramer procedure (R, lsmeans package).

After DNA extraction following a conventional phenol/chloroform procedure, the sex of selected animals was verified by PCR using primers specific for chicken sex chromosome W^43^. Individual samples of duodenum (*n* = 12), jejunum (*n* = 12), ileum (*n* = 12), and breast muscle (*n* = 12) were used for RNA isolation. Extraction of total RNA was performed according to TRIzol Reagent protocol (Invitrogen, Darmstadt, Germany). Therefore, samples were disrupted using either mortar and pestle (duodenum, jejunum, and ileum) or a Precellys cell disruption device (breast muscle; 12 sec at 5000U; Peqlab, Erlangen, Germany). Subsequently, total RNA was treated with Baseline-ZERO DNase (Biozym, Hessisch Oldendorf, Germany). Purification of RNA was achieved using the NucleoSpin RNAII kit (Macherey-Nagel, Düren, Germany) according to manufacturer’s protocols. Total RNA was checked on agarose gel and measured by NanoDrop ND-1000 (NanoDrop, Peqlab, Erlangen, Germany). The quality of RNA was assessed employing an Agilent 2100 Bioanalyzer (Agilent Technologies, Santa Clara, CA, USA). All samples had RNA integrity numbers (RIN) values ≥ 7.8. Subsequent extraction of mRNA was supported by a magnetic mRNA Isolation Kit (New England Biolabs, Frankfurt, Germany). Libraries were prepared using the TruSeq Stranded mRNA preparation kit (Illumina, San Diego, CA, USA). Quantity of libraries were analysed using both a DNA 1000 chip on a Bioanalyzer instrument and the Illumina Library Quantification Kit (Kapa Biosystems, Cape Town, South Africa) performed on a LightCycler 480 PCR System (Roche, Mannheim, Germany). Quality controlled libraries were sequenced on an Illumina HiSeq. 2500 instrument (Illumina) by generating 125-bp paired-end reads in high-output run mode as described by the manufacturer.

### Transcript data analyses

After demultiplexing, adapter-trimming, and base-quality filtering, TopHat (version 2.0.12) and Bowtie (version 1.1.1.0) mapping tools were used to align the sequences to the chicken reference genome (*Gallus gallus* genome build 5, March 2017). Read counts per gene were calculated via htseq-count version 0.6.1p1^44^. In order to exclude genes with very low read counts, features with less than 5 reads in at least 2 samples were discarded from further analyses. Quality of sequencing data was assessed using the arrayQualityMetrics package for R (version 3.32.0). The package uses three different outlier detection approaches, including the distance between individual data sets, the distribution of signal intensities and the quality of the individual data set. Samples were judged as outliers which were conspicuous in at least two of these criteria. In total, 4 out of 48 samples (4 tissues, *n* = 12) were removed from further analyses (see Supplementary Fig. S1).

Analysis of differentially expressed genes was performed using the DESeq. 2 package of the R environment^45^. For each tissue and sex, the negative binomial generalised linear model included day of slaughter and RFI group as fixed effects. Corresponding contrasts were assessed via Wald tests. False discovery rates (FDR, q-values) were calculated using the qvalue R package following the Benjamini-Hochberg (BH) procedure (version 2.8.0). The significance threshold was set at p < 0.001 and q < 0.25. Fold changes were calculated to display the relative transcript abundance between high FE and low FE groups (high FE > low FE indicated by positive FC; high FE < low FE indicated by negative FC). No FC cut off was applied. The number of differentially abundant transcripts and the size of the intersections between experimentally groups were displayed using the UpSetR package^46^.

### Analysis of canonical pathways and biofunctions

Lists of significantly altered transcripts were evaluated with Ingenuity Pathway Analysis (IPA; Ingenuity Systems, Redwood City, CA). Human gene identifiers were preferentially used if available. Therefore, orthologous gene identifiers were assigned using the BiomaRt R package (Gallus_gallus-5.0 and GRCh38.p7, version 88, accessed on March 2017). Results related to canonical pathways and biofunctions were considered significant at a Benjamini-Hochberg adjusted p-value < 0.05. In addition, molecular themes related to biofunctions had to pass a second cut-off criteria (absolute value of z-scores > 2.0 were considered as significant) stating a prediction of its activation status^47^.

### Quantitative real-time PCR (qPCR)

First-strand cDNA was synthesized from 1.5 µg of total RNA using random primers and oligo d(T) 13VN in the presence of Superscript III reverse transcriptase (Invitrogen, Karlsruhe, Germany) and RNAsinPlus RNase Inhibitor (Promega, Heidelberg, Germany). The cDNA samples were diluted in 200 µL Aqua dest. and stored at −20 °C until further analyses. Transcript levels of selected target (*ACSBG2*, *AMY2A*, *AQP4*, *CA7*, *FABP4*, *FNDC5*, *G6PC*, *GATM*, *GBP*, *HPX*, *PFKFB*, *THRSP*) and reference genes (*ACTB*, *GAPDH*, *DCAF7*) were quantified by qPCR using transcript-specific primers. Primer specifications and tissues analysed are shown in Supplementary Table S2. Individual mRNA samples (n = 12 per tissue) were analysed in duplicate on a LightCycler 480 system using the LightCycler 480 SYBR Green I Master (Roche, Mannheim, Germany) according to manufacturer’s instructions. Amplified products were subjected to melting curve analyses and gel electrophoresis to verify the absence of non-specific products. For all assays, threshold cycles were converted to copy numbers using a standard curve generated by amplifying serial dilutions of a corresponding PCR standard (10^7^–10^2^ copies). Values were calculated by factorial normalisation on *ACTB*, *GAPDH*, and *DCAF7* expression values. Correlation of normalized expression values was calculated using Pearson test and considered significant at p < 0.05.

## Electronic supplementary material

Table S1

Table S2, Table S3, Figure S1

## Data Availability

The data generated during the current study have been deposited in the ArrayExpress database at EMBL-EBI (www.ebi.ac.uk/arrayexpress) under accession number E-MTAB-6169.
