# Supplementary material for: Transcriptional shifts account for divergent resource allocation in feed efficient broiler chickens
Source: Sci Rep. 2018 Aug 27;8:12903. doi: 10.1038/s41598-018-31072-7 (PMC6110741; doi:10.1038/s41598-018-31072-7)
Supplement: Supplementary file 2 — Table S2, Table S3, Figure S1 [file 41598_2018_31072_MOESM2_ESM.pdf]

Transcriptional shifts account for divergent resource allocation in feed efficient broiler chickens

Henry Reyer, Barbara U. Metzler-Zebeli, Nares Trakooljul, Michael Oster, Eduard Murani, Siriluck Ponsuksili, Frieder Hadlich, and Klaus Wimmers

Supplementary Table S2. Primers used in qRT-PCR to verify RNA-seq results

Primer	Sequence	Melting temp. (°C)	Amplicon length (bp)	Fragment type	Tissue[1]
Gga_ACTB_f1	CCTCTTCCAGCCATCTTTCTT	57.9	254	qRT-PCR/ Standard	Mu, Duod, Jeju, Ile
Gga_ACTB_r1	TAGAGCCTCCAATCCAGACA	55.3			
Gga_ACSBG2_f	AATTCCTTGCCATGCTTCTAAC	58.3	154	qRT-PCR/ Standard	Mu
Gga_ACSBG2_r	GCCTTGTCTTTGCTGCTTATG	58.1			
Gga_AMY2A_f1	CAAACTGCACGATCTAAATACTCAG	58.5	189	qRT-PCR	Duod
Gga_AMY2A_r1	GCCATCTTCTCTCCATTCCAC	58.5			
Gga_AMY2A_f2	TGATATGGGTGTAGCAGGGTTC	59.2	348	Standard	
Gga_AMY2A_r2	CGCTGGTTGTCGTGGTTATC	58.7			
Gga_AQP4_f1	CTCATTTGGACCTGCTGTCATC	59.5	179	qRT-PCR	Mu
Gga_AQP4_r1	TCCCTTTGGATGGCTGAGTAG	59.3			
Gga_AQP4_f2	GTGCCAGTATGAACCCTGCTC	59.5	373	Standard	
Gga_AQP4_r2	TCTCTGCTTTCAGTGCTCCTTG	59.9			
Gga_CA7_f1	AGATGGCTTGGCTGTAGTTGG	59.4	141	qRT-PCR/ Standard	Duod, Ile
Gga_CA7_r1	AGGAGACATTTGGGGTTGAAG	58.2			
Gga_DCAF7_f1	GTACTTCTAGCATTGACACAACCTG	58.1	166	qRT-PCR	Mu, Duod, Jeju, Ile
Gga_DCAF7_r1	AACATATCTCTCCCACCACCTG	58.1			
Gga_DCAF7_f2	GTGGAGAGTGGGTGAAACAGAG	58.5	556	Standard	
Gga_DCAF7_r2	AATGTGGCAGGAAGAATGAGG	59.1			
Gga_FABP4_f1	TGATGAGACCACAGCAGATGAC	58.0	117	qRT-PCR	Jeju
Gga_FABP4_r1	CCACCACTTTTCTCTTGATAACAG	58.0			
Gga_FABP4_f2	CTGGGTGTGGGGTTTGCTAC	60.1	338	Standard	
Gga_FABP4_r2	CGGCTTCCTCATGCTCTTTC	59.9			
Gga_FNDC5_f1	ATGGCGAAGAAAAACCAACAG	59.6	119	qRT-PCR	Mu
Gga_FNDC5_r1	TTGTCTTTGATGATGTCGTACTGC	59.7			
Gga_FNDC5_f2	TTGTCATTGGATTTGCCATTTC	60.1	323	Standard	
Gga_FNDC5_r2	GGCTTTCTCCTTGCTGTTGTTC	60.8			
Gga_G6PC_f	TCCATTTGTGTGAGCCAGTG	57.1	144	qRT-PCR/ Standard	Mu, Jeju
Gga_G6PC_r	CAGAGGCGTTGCTGTAATAATC	57.3			
Gga_GAPDH_f1	AGTCGGAGTCAACGGATTTG	57.4	251	qRT-PCR/ Standard	Mu, Duod, Jeju, Ile
Gga_GAPDH_r1	CTGCCCATTTGATGTTGCTG	59.7			
Gga_GATM_f1	GATCCAAACCCTATGCACATTG	59.7	185	qRT-PCR	Mu
Gga_GATM_r1	GAAAGCCATTTAGAAGACATCCAC	59.4			
Gga_GATM_f2	TCCTATCCGCTCTGTTGAAGAC	58.7	433	Standard	
Gga_GATM_r2	CATCACACGCTTCTCATCCAG	58.7			
Gga_GBP_f1	TACATCGGGACTGTGAGCAAC	58.3	125	qRT-PCR/ Standard	Duod, Ile
Gga_GBP_r1	CTGGCAGAACTCCTCTTTGG	57.3			

Supplementary Table S2. Continued.

Primer	Sequence	Melting temp. (°C)	Amplicon length (bp)	Fragment type	Tissue[1]
Gga_HPX_f1	CACCCAGTTCTATCGGTTCAG	57.2	141	qRT-PCR	Mu
Gga_HPX_r1	CAGCGGTCACCAGCATTG	58.4			
Gga_HPX_f2	TCAAGGGGGATAAGGTGTTCTC	59.8	429	Standard	
Gga_HPX_r2	AGAAGGCAGCATCCACATCAC	59.8			
Gga_PFKFB3_f1	AATGCCCTACCTGAAATGTCC	58.3	148	qRT-PCR/ Standard	Jeju
Gga_PFKFB3_r1	CGGGTTAGGTCCCTTCTTTG	58.9			
Gga_THRSP_f1	GCAGACCTCTACGAGCACTACC	58.5	148	qRT-PCR	Jeju
Gga_THRSP_r1	GATTCCCATCTATGCCATCTATG	58.2			
Gga_THRSP_f2	ATGGAGCAGGAGGTGATGTTC	58.7	302	Standard	
Gga_THRSP_r2	GCTTTGGTGTTTTTGGTGAGG	59.8			

ACTB – Actin beta; *ACSBG2* – Acyl-CoA Synthetase Bubblegum Family Member 2); *AMY2A* – Amylase, Alpha 2A (Pancreatic); *AQP4* – Aquaporin 4; *CA7* – Carbonic Anhydrase 7; *DCAF7* – DDB1 and CUL4 Associated Factor 7; *FABP4* – Fatty Acid Binding Protein 4; *FNDC5* – Fibronectin Type III Domain Containing 5; *G6PC* - Glucose-6-Phosphatase Catalytic Subunit; *GAPDH* – Glyceraldehyde-3-Phosphate Dehydrogenase; *GATM* – Glycine Amidinotransferase; *GBP* – Guanylate Binding Protein; *HPX* – Hemopexin; *PFKFB3* – 6-Phosphofructo-2-Kinase/Fructose-2,6-Biphosphatase 3; *THRSP* – Thyroid Hormone Responsive

[1] Tissues analyzed by qPCR to verify results of RNA-seq; Mu – muscle, Duod – duodenum, Jeju – jejunum, Ile – ileum

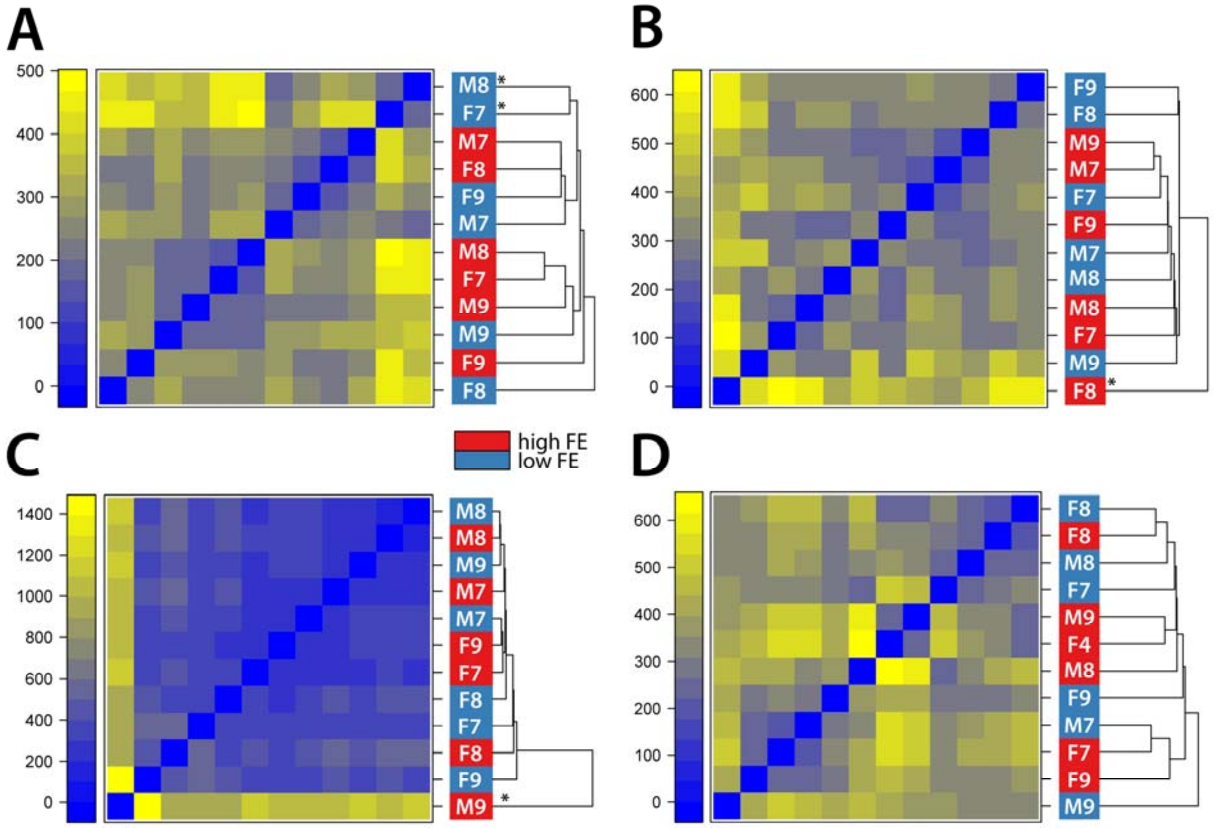

Supplementary Figure S1

Heatmaps representing distances between individual RNA-seq results of analysed male (M) and female (F) chickens divergent for feed efficiency (A - breast muscle, B - duodenum, C - jejunum and D - ileum). Unique animal identifiers are indicated. Samples marked with an asterisk were removed from further analysis.

Table S3. Correlation of RNA-seq and quantitative real-time PCR (qRT-PCR) results for selected transcripts.

Gene symbol (sex)	Ensembl ID	Correlation	
		**p-value**	**Coefficient**
Breast muscle			
ACSBG2 (male)	ENSGALG00000001749	0.110	0.795
AQP4 (male)	ENSGALG00000015128	0.340	0.547
FNDC5 (male)	ENSGALG00000003567	0.005	0.976
GATM (male)	ENSGALG00000023435	0.015	0.947
G6PC (male)	ENSGALG00000030034	0.094	0.813
HPX (male)	ENSGALG00000022586	0.055	0.870
ACSBG2 (female)	ENSGALG00000001749	0.256	0.629
AQP4 (female)	ENSGALG00000015128	0.023	0.929
FNDC5 (female)	ENSGALG00000003567	0.020	0.934
GATM (female)	ENSGALG00000023435	<0.001	0.992
G6PC (female)	ENSGALG00000030034	0.135	0.762
HPX (female)	ENSGALG00000022586	0.023	0.928
Duodenum			
AMY2A (male)	ENSGALG00000038740	0.028	0.861
CA7 (male)	ENSGALG00000033720	0.010	0.918
GBP (male)	ENSGALG00000026152	0.054	0.803
AMY2A (female)	ENSGALG00000038740	<0.001	0.999
CA7 (female)	ENSGALG00000033720	0.005	0.976
GBP (female)	ENSGALG00000026152	0.291	0.594
Jejunum			
FABP4 (male)	ENSGALG00000030025	0.008	0.965
G6PC (male)	ENSGALG00000030034	0.021	0.933
PFKFB3 (male)	ENSGALG00000040369	0.028	0.917
THRSP (male)	ENSGALG00000035803	0.003	0.982
FABP4 (female)	ENSGALG00000030025	<0.001	0.985
G6PC (female)	ENSGALG00000030034	0.129	0.690
PFKFB3 (female)	ENSGALG00000040369	0.092	0.742
THRSP (female)	ENSGALG00000035803	0.002	0.964
Ileum			
CA7 (male)	ENSGALG00000033720	0.011	0.956
GBP (male)	ENSGALG00000026152	0.405	0.488
CA7 (female)	ENSGALG00000033720	0.016	0.847
GBP (female)	ENSGALG00000026152	0.311	0.501
